# From ER to OR: Results After Implementation of Multidisciplinary Pathway for Fragility Hip Fractures at a Level I Trauma Center

**DOI:** 10.1177/2151459320927383

**Published:** 2020-05-22

**Authors:** Kenoma Anighoro, Carla Bridges, Alexander Graf, Alexander Nielsen, Tannor Court, Jack McKeon, Joseph M. Schwab

**Affiliations:** 1 Department of Orthopedic Surgery, Medical College of Wisconsin, Milwaukee, WI, USA

**Keywords:** fragility fractures, geriatric trauma, osteoporosis, systems of care, trauma surgery

## Abstract

**Introduction:**

Hip fractures are one of the most common indications for hospitalization and orthopedic intervention. Fragility hip fractures are frequently associated with multiple comorbidities and thus may benefit from a structured multidisciplinary approach for treatment. The purpose of this article was to retrospectively analyze patient outcomes after the implementation of a multidisciplinary hip fracture pathway at a level I trauma center.

**Materials and Methods:**

A retrospective review of 263 patients over the age of 65 with fragility hip fracture was performed. Time to surgery, hospital length of stay, Charlson Comorbidity Index (CCI), American Society of Anesthesiologists, complication rates, and other clinical outcomes were compared between patients treated in the year before and after implementation of a multidisciplinary hip fracture pathway.

**Results:**

Timing to OR, hospital length of stay, and complication rates did not differ between pre- and postpathway groups. The postpathway group had a greater CCI score (pre: 3.10 ± 3.11 and post: 3.80 ± 3.18). Fewer total blood products were administered in the postpathway group (pre: 1.5 ± 1.8 and post: 0.8 ± 1.5).

**Discussion:**

The maintenance of clinical outcomes in the postpathway cohort, while having a greater CCI, indicates the same quality of care was provided for a more medically complex patient population. With a decrease in total blood products in the postpathway group, this highlights the economic importance of perioperative optimization that can be obtained in a multidisciplinary pathway.

**Conclusion:**

Implementation of a multidisciplinary hip fracture pathway is an effective strategy for maintaining care standards for fragility hip fracture management, particularly in the setting of complex medical comorbidities.

## Introduction

Fragility hip fractures are one of the most common indications for hospitalization and orthopedic intervention. Each year in the United States alone, an estimated 300 000 hip fracture hospitalizations are reported. It is predicted that by 2040 there may be up to 840 000 per year, with the majority requiring surgical treatment.^1,2^ Patients with fragility hip fractures have unique needs when compared to the typical orthopedic trauma patient. Besides abnormal bone mineral density and advanced age, this patient population is also prone to frailty, comorbid medical conditions, and poor surgical outcomes, indicating a need for improved care across multiple medical specialties.^3^ Current standard of care for hip fracture treatment in the elderly population is to administer surgical intervention within 48 hours of injury after medical optimization to improve clinical outcomes and reduce mortality.^4^ To achieve this level of care, standardized multidisciplinary health care pathways have been developed to streamline the flow of care from initial evaluation in the emergency room (ER) to surgical intervention in the operating room (OR). The goal of these pathways is to improve care quality and efficiency by minimizing variability in service delivery.^5^

Previous studies have shown that implementation of a standardized multidisciplinary pathway for the treatment of fragility hip fractures in the elderly population improves care and decreases hospital length of stay, postoperative complications, and mortality.^6-8^ By identifying the mandatory steps between the ER and OR, as well as potential modifiable risk factors, activation of a standardized pathway enables multiple health care providers to work in parallel rather than sequentially to create an efficient, predictable high level of care. The roles of various providers and operational variables that constitute an institutional multidisciplinary hip fracture pathway have been well described in the literature.^9^ However, the key elements include prompt diagnosis through streamlined diagnostic imaging in the ED, preoperative medical and anesthesia evaluation, and a multimodal pain control regimen using neuraxial anesthesia to minimize narcotic use for optimized perioperative outcomes.^10-12^ The goal of our study was to compare perioperative outcomes in elderly patients who underwent hip fracture treatment at our level I trauma center before and after implementation of a standardized multidisciplinary pathway in order to understand its effect and ultimately improve care for this common and often medically complex patient population.

## Materials and Methods

**Figure 1.**
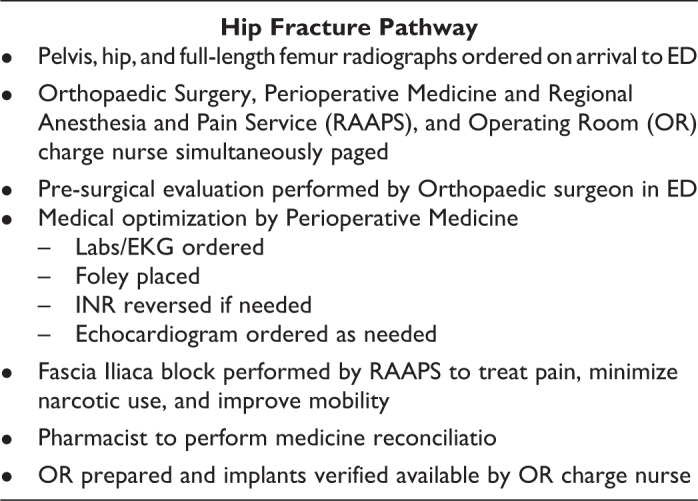
Detailed description of hip fracture pathway.

A retrospective cohort study of patients over 65 years of age diagnosed with a fragility hip fracture was performed on patients admitted to our level I trauma hospital 1 year before and after implementation of a multidisciplinary hip fracture pathway (Figure 1). Patients were included if they had an isolated femoral neck, intertrochanteric, or subtrochanteric fracture sustained through a low-energy mechanism and the patient and/or his or her power of attorney desired surgical treatment. Periprosthetic fractures, pathologic fractures, patients with high-energy mechanisms with associated acetabular fractures, and polytrauma patients were excluded. For the prepathway group, 116 patients met the inclusion criteria. For the postpathway group, 147 patients were identified who met the inclusion criteria and were admitted after 1 year had passed since implementation of the pathway to avoid influence of logistical irregularities due to gradual onboarding of the pathway. Data collected included age, sex, American Society of Anesthesiologists (ASA) classification, body mass index (BMI), Charlson Comorbidity Index (CCI), fracture type, surgery type, time to surgery, length of stay, admission hemoglobin and hematocrit, number of blood products used during admission, intensive care unit (ICU) admission, and incidence of postoperative complications. These patient characteristics were compared across 2 groups (pre vs post) either using *t* test or Wilcoxon sum test for continuous variables and using χ^2^ test for categorical variables. Exact versions of Wilcoxon sum and χ^2^ tests were used to reduce the impact of small sample sizes.

## Results

Pre- and postpathway patient demographics were similar, with no statistically significant differences with regard to age, sex, BMI, fracture type, or surgery performed (Table 1). The majority of patients were female in both groups, with 63.8% in the prepathway group and 74.8% in the postpathway group (*P* = .053). The average patient age in the 2 groups was 82 and 83 years, respectively (*P* = .712). The most common ASA class across both the pre- and postpathways was ASA class III, 64.7% (pre) and 68.7% post (*P* = .677). The average CCI was 3.10 ± 3.11 (pre) and 3.80 ± 3.18 (post) (*P* = .043). Average time to surgery was 0.89 days (pre) versus 0.75 days (post) (*P* = .20). The percentage of patients receiving surgery in <24 hours was 63.8% (pre) and 72.8% (post) (*P* = .12); the percentage of patients receiving surgery in <48 hours was 92.2% (pre) and 93.2% (post) (*P* = 0.77; Table 2). Length of stay was 6.6 ± 4.4 days (pre) versus 7.1 ± 6.7 days (post) (*P* = .25). Total blood product units given during admission were 1.5 ± 1.8 (pre) and 0.8 ± 1.5 (post) (*P* = .002). Average number of packed red blood cell units transfused was 1.0 (pre) and 0.8 (post) (*P* = .124). Average fresh frozen plasma (FFP) units administered was 0.13 ± 0.61 (pre) and 0.02 ± 0.18 (post) (*P* = .077). Average number of platelet units administered was 0.026 ± 0.16 (pre) and 0.22 ± 0.02 (post) (*P* = .389). Thirty-day mortality was 6.9% (pre) and 2% (post) (*P* = .085). Thirty-day readmissions were 10.3% (pre) and 10.9% (post) (*P* = .428). Postoperative ICU admissions were 18.1% (pre) and 11% (post) (*P* = .11). The number of patients with any postoperative complication was 46.6% (pre) and 47.6% (post) (*P* = 0.86). Mortality was analyzed at 30 days being 6.0% (pre) and 2.0% (post) (*P* = .0644). There was 1 patient in the prepathway group who was lost to follow-up.

**Table 1.** Patient Demographics of Pre- and Postpathway Patient Populations.

Variables	Group			*P* value
	Total, N = 263(col %)	Pre, n = 116 (col %)	Post, n = 147(col %)	
Age at admission				.712
Mean ± SD	83 ± 9	82 ± 9	83 ± 9	
Gender				.053
Female	184 (70.0)	74 (63.8)	110 (74.8)	
Male	79 (30.0)	42 (36.2)	37 (25.2)	
BMI				.1308
Underweight	22 (8.4)	10 (8.6)	12 (8.2)	
Normal	126 (47.9)	49 (42.2)	77 (52.4)	
Overweight	72 (27.4)	37 (31.9)	35 (23.8)	
Class 1 obesity	29 (11.0)	14 (12.1)	15 (10.2)	
Class 2 obesity	7 (2.7)	4 (3.4)	3 (2.0)	
Class 3 obesity	7 (2.7)	2 (1.7)	5 (3.4)	
ASA				.677
2	19 (7.2)	10 (8.6)	9 (6.1)	
3	176 (66.9)	75 (64.7)	101 (68.7)	
4	68 (25.9)	31 (26.7)	37 (25.2)	
Surgery				.775
Intramedullary nail	144 (54.8)	61 (52.6)	83 (56.5)	
Hip hemiarthroplasty	102 (38.8)	47 (40.5)	55 (37.4)	
Total hemiarthroplasty	11 (4.2)	6 (5.2)	5 (3.4)	
Open reduction internal fixation	6 (2.3)	2 (1.7)	4 (2.7)	
Fracture				.643
Femoral neck	114 (43.3)	54 (46.6)	60 (40.8)	
Intertrochanteric	128 (48.7)	53 (45.7)	75 (51.0)	
Subtrochanteric	21 (8.0)	9 (7.8)	12 (8.2)	
Hgb				.917
Mean ± SD	12.15 ± 1.73	12.14 ± 1.54	12.16 ± 1.88	
HCT				.464
Mean ± SD	36.94 ± 4.92	36.69 ± 4.40	37.14 ± 5.30	
Charlson Comorbidity Index (CCI)				.043
Mean ± SD	3.49 ± 3.16	3.10 ± 3.11	3.80 ± 3.18	

Abbreviations: ASA, American Society of Anesthesiologists; BMI, body mass index; Hgb, hemoglobin; HCT, hematocrit.

**Table 2.** Clinical Outcomes of Pre- and Postpathway Patient Populations.

Variables	Group			*P* value
	Total, N = 271 (col %)	Pre, n = 116 (col %)	Post, n = 147 (col %)	
Time to surgery (TTS), days				.8939
Mean ± SD	0.96 ± 0.71	0.98 ± 0.67	0.95 ± 0.74	
Length of stay (LOS), days				.0254
Mean ± SD	6.87 ± 5.76	6.57 ± 4.36	7.10 ± 6.65	
ICU admission (total)				
Admitted	36 (13.3)	21 (18.1)	15 (10.2)	
FFP (units)				
0	255 (97.0)	110 (94.8)	145 (98.6)	
1	2 (0.8)	1 (0.9)	1 (0.7)	
2	4 (1.5)	3 (2.6)	1 (0.7)	
4	2 (0.8)	2 (1.7)	0 (0.0)	
Platelets (units)				
0	258 (98.1)	113 (97.4)	145 (98.6)	
1	4 (1.5)	3 (2.6)	1 (0.7)	
2.5	1 (0.4)	0 (0.0)	1 (0.7)	
Complications (total)				
0	148 (56.3)	70 (60.3)	78 (53.1)	
1	77 (29.3)	28 (24.1)	49 (33.33)	
2	27 (10.3)	13 (11.2)	14 (9.5)	
3	9 (3.4)	5 (4.3)	4 (2.7)	
4	2 (0.8)	0 (0.0)	2 (1.4)	
30-day mortality (total)				
Deceased	10 (3.7)	7 (6.0)	3 (2.0)	
30-day readmission (total)				
Readmission	27 (10.4)	9 (7.8)	16 (10.9)	

Abbreviations: FFP, fresh frozen plasma; ICU, intensive care unit.

## Discussion

Fragility hip fractures in the elderly population are common and have been shown previously to require an efficient, multidisciplinary approach in order to optimize patient outcomes. However, after implementation of our multidisciplinary pathway, we did not find that patients got to the OR sooner, had lower complication rates, or had decreased length of stays when compared to the cohort analyzed just prior to implementation of the pathway. However, we did find that the post-athway group had higher levels of comorbidity (as evidenced by CCI) and received fewer total blood products perioperatively. The former is likely secondary to the increased volume of patients transferred from outside hospitals within our health care system during the study period. The lower incidence of blood products could be attributable to early recognition of need for international normalized ratio reversal by medicine colleagues and therefore the decision to use vitamin K versus FFP or an increased incidence of intraoperative tranexamic acid use in the postpathway group. However, due to the low overall incidence of blood product administration and small difference between the 2 groups (0.8 and 1.5 units on average, respectively), this is unlikely a clinically significant difference.

Although the majority of clinical outcomes did not differ between pre- and postpathway groups, one important distinction between the groups is the higher patient volume and CCI in the postpathway treatment group. Overall, these similar outcomes between groups, with over 92% of patients receiving surgery at the target of 48 hours or less after injury, support the notion that a systematic pathway for treating patients with fragility hip fractures is effective and can consistently supply appropriate care to this patient population despite higher comorbidity and patient volume. It is impossible to determine whether the postpathway patient population would have fared the same regardless of pathway utilization due to the nature of the change. To completely determine the effectiveness of the change to a multidisciplinary pathway, a patient control group could have been utilized, but due to facility restraints and limitations, this was not possible. In addition, the patient population in the postpathway group would have been limited if a control group was utilized.

Limitations of this study include the retrospective nature of the review as well as other bottlenecks to efficient hip fracture care beyond the authors’ control, including inpatient bed availability, high acuity patient demand in the emergency department, resource-intensive diagnostic testing (eg, echocardiography), consultant bandwidth, and OR availability. At the same time of protocol initiation, our facility implemented a “no divert” policy preventing certain patient transfers to other facilities. Facility changes like this further affect patient volume and bed availability. For our facility, none of these effects could be predicted at the onset of pathway implementation and individual changes like this could not be accounted for in our analysis.

An important benefit to the hip fracture pathway that is not captured by our data is the subjective improvement in many qualitative changes to patient care, including clinical workflow and provider burden that are all positively affected by implementation of this pathway. By transitioning to a more team-based philosophy, standardizing the preoperative workup, and clearly defining the roles of each team member, more efficient interprovider communication and patient evaluation occurred, resulting in less interprovider friction, errors in handoffs, as well as more opportunities for parallel processing.

## Conclusion

Implementation of a multidisciplinary health care pathway for fragility hip fractures helps to maintain good clinical outcomes despite increased comorbidity burden and patient volume. Further studies are needed to identify how to best utilize multidisciplinary hip fracture pathways to streamline quality care for this ever-increasing and medically complex patient population.
